# Functional neural differentiation of human adipose tissue-derived stem cells using bFGF and forskolin

**DOI:** 10.1186/1471-2121-11-25

**Published:** 2010-04-16

**Authors:** Sujeong Jang, Hyong-Ho Cho, Yong-Bum Cho, Jong-Seong Park, Han-Seong Jeong

**Affiliations:** 1 Department of Physiology, Chonnam National University Medical School, Gwangju 501190, Republic of Korea; 2 Department of Otolaryngology, Chonnam National University Medical School, Gwangju 501190, Republic of Korea; 3 Brain Korea 21 Project, Center for Biomedical Human Resources at Chonnam National University, Gwangju, 501-190, Republic of Korea; 4 Research Institute of Medical Sciences, Chonnam National University, Gwangju 501-190, Republic of Korea

## Abstract

**Background:**

Adult mesenchymal stem cells (MSCs) derived from adipose tissue have the capacity to differentiate into mesenchymal as well as endodermal and ectodermal cell lineage *in vitro*. We characterized the multipotent ability of human adipose tissue-derived stem cells (hADSCs) as MSCs and investigated the neural differentiation potential of these cells.

**Results:**

Human ADSCs from earlobe fat maintained self-renewing capacity and differentiated into adipocytes, osteoblasts, or chondrocytes under specific culture conditions. Following neural induction with bFGF and forskolin, hADSCs were differentiated into various types of neural cells including neurons and glia *in vitro*. In neural differentiated-hADSCs (NI-hADSCs), the immunoreactivities for neural stem cell marker (nestin), neuronal markers (Tuj1, MAP2, NFL, NFM, NFH, NSE, and NeuN), astrocyte marker (GFAP), and oligodendrocyte marker (CNPase) were significantly increased than in the primary hADSCs. RT-PCR analysis demonstrated that the mRNA levels encoding for ABCG2, nestin, Tuj1, MAP2, NFL, NFM, NSE, GAP43, SNAP25, GFAP, and CNPase were also highly increased in NI-hADSCs. Moreover, NI-hADSCs acquired neuron-like functions characterized by the display of voltage-dependent tetrodotoxin (TTX)-sensitive sodium currents, outward potassium currents, and prominent negative resting membrane potentials under whole-cell patch clamp recordings. Further examination by RT-PCR showed that NI-hADSCs expressed high level of ionic channel genes for sodium (SCN5A), potassium (MaxiK, Kv4.2, and EAG2), and calcium channels (CACNA1C and CACNA1G), which were expressed constitutively in the primary hADSCs. In addition, we demonstrated that Kv4.3 and Eag1, potassium channel genes, and NE-Na, a TTX-sensitive sodium channel gene, were highly induced following neural differentiation.

**Conclusions:**

These combined results indicate that hADSCs have the same self-renewing capacity and multipotency as stem cells, and can be differentiated into functional neurons using bFGF and forskolin.

## Background

Stem cell-based therapies for the repair and regeneration of various tissues and organs offer a paradigm shift that may lead to alternative therapeutic solutions for a number of diseases. The emerging field of regenerative medicine requires reliable sources of stem cells, biomaterial scaffolds and cytokine growth factors. A stem cell is characterized by its ability to self-renew and to differentiate along multiple lineage pathways. The multi-lineage potentials of embryonic stem cells (ESCs) and adult stem cells from bone marrow have been extensively documented [1-4]. Although ESCs have been isolated from humans and have enormous potential, their use in therapeutics is restricted by ethical and political issues [5,6]. Compared to ESCs, adult stem cells have diminished self-renewal capability and multipotency. By nature, however, adult stem cells are immunocompatible and there are no ethical issues related to their use. The advantage of using mesenchymal stem cells (MSCs) over other cell types is the ability to take advantage of their autologous properties [7-11]. MSCs can easily be obtained from the patient's own tissue, including bone marrow, adipose tissue, cartilage, synovium, periosteum, muscle, and palatine tonsil and expanded vigorously until the tissues differentiate into specific cell lineages [12-21].

Neural tissue is understood to have a limited capacity for repair after injury, and adult neurogenesis is limited to selected regions of the brain, including the hippocampus, the subventricular zone, and the olfactory system [22,23]. Therefore, a broad spectrum of cells capable of neuronal differentiation is required for cell replacement therapies. Adult peripheral tissues may be an alternative source of stem and progenitor cells. For example, a number of studies have shown that adult bone marrow contains a population of mesenchymal stem cells capable of differentiating into several lineages, including neuronal and glial tissues [2,13,24-29]. However, bone marrow procurement is extremely painful for patients and yields low numbers of harvested cells.

Adipose tissue is emerging as a source of stem cells obtained by less invasive methods including lipoaspiration, and in larger quantities than bone marrow. Adipose tissue, like bone marrow, is derived from the embryonic mesoderm and contains a heterogeneous stromal cell population that includes mature adipocytes, preadipocytes, fibroblasts, vascular smooth muscle cells, endothelial cells, monocyte/macrophages, and lymphocytes [30]. Adipose tissue-derived stem cells (ADSCs) are self-renewing and can differentiate along several mesenchymal tissue lineages, including adipocytes, osteoblasts, myocytes, chondrocytes, endothelial cells, and cardiomyocytes [14,31-35]. Since human adipose tissue is ubiquitous and easily obtained in large quantities under local anesthesia with little patient discomfort, it may present an alternative source of stem cells for mesenchymal tissue regeneration and engineering.

Recent studies report that ADSCs can also be induced into neuron- or glia-like cells *in vitro *[12,20,36-40], but whether MSCs can actually differentiate into neurons and glia remains controversial [41,42]. The neuron-like morphology and immunocytochemical expression for neural markers in transdifferentiated bone marrow-derived MSCs under culture or *in vivo *conditions may be due to cell fusion [43-45]. On the contrary, some investigators have demonstrated that neural differentiation is induced independently of cell fusion [46], indicating either that MSCs have an intrinsic ability for differentiation beyond their organ of residence or that MSC differentiation potential can be reprogrammed toward specific cell lineages by exogenous cues [47]. These results imply that the capacity of ADSCs for neural differentiation should be evaluated functionally and morphologically.

This study aimed to examine whether neurally-induced hADSCs (NI-hADSCs) displayed the functional characteristics of neural cells. We have characterized the multipotent capacity of hADSCs isolated from earlobe fat and investigated the differentiation potential of stem cells into neural cells in the presence of bFGF and forskolin, which are known to enhance the neuronal differentiation of bone marrow-derived MSCs [26] over two weeks. We identified that NI-hADSCs displayed voltage-dependent and TTX-sensitive sodium currents, which are a functional hallmark of neurons, and expressed high levels of ionic channel genes, which are important in neural function.

## Results

### Isolation and characterization of adipose tissue-derived stem cells

**Figure 1:**
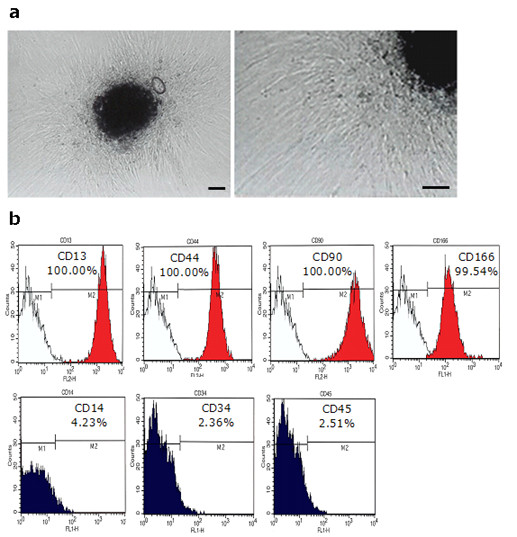
**Isolation and characterization of hADSCs**. **a**, Human ADSCs were isolated from fat tissue according to their adherence to culture dishes. Under phase contrast, hADSCs are spindle-shaped after seven days in culture. Scale bars measure 50 μm. **b**, Human ADSCs were labeled with FITC-conjugated antibodies specific to CD14, CD34, and CD45. Human ADSCs were labeled with PE-conjugated antibodies specific to CD13, CD44, CD90, and CD166. Surface phenotype was analyzed by FACS. Open histograms refer to control immunoglobulins and colored histograms refer to specific antibodies. Primary hADSCs expressed MSC specific markers including CD13 (+), CD44 (+), CD90 (+), and CD166 (+), but did not express hematopoietic stem cell markers including CD14 (-), CD34 (-), and CD45 (-).

Human mesenchymal stem cells were isolated from earlobe fat according to their adherence to culture dishes containing DMEM supplemented with 10% FBS. The cells were expanded after plating and grown to confluence. Approximately four weeks into the culture, cells became more uniform and grew in a monolayer with typical fibroblast-like morphology (Figure 1a). The proliferation of human adipose tissue-derived stem cells (hADSCs) remained consistent up until the 15th passage.

To clarify the isolated hADSCs, we performed FACS analysis with various cell surface markers, including MSC-specific cell type markers and hematopoietic stem cell markers (Table 1). As shown in Figure 1b, more than 95% of the adipose tissue-derived hMSCs expressed MSC-specific markers, including CD13, CD44 (endoglin), CD90 (Thy-1), and CD166, but did not express markers for hematopoietic stem cells, including CD14, CD34, and CD45. Thus, hADSCs in this experiment appeared to be MSCs.

**Table 1:** Phenotypic analysis of hMSCs with FACS

Antigen	Description	(+)/(-)
CD13	Bone marrow stromal cell marker	(+)

CD44	Endoglin receptor	(+)

CD90 (Thy-1)	Endothelial cell marker	(+)

CD166	Cell adhesion molecule	(+)

CD14	Lipopolysaccharide receptor	(-)

CD34	Hematopoietic progenitor cell marker	(-)

CD45	Hematopoietic progenitor cell marker	(-)

### Pluripotent capability of hADSCs

**Figure 2:**
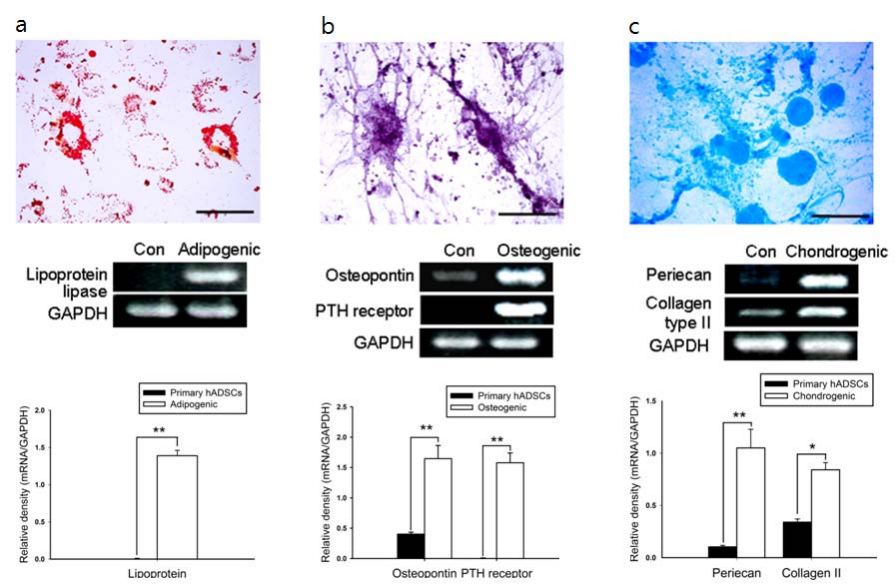
**Investigation of the multipotent capacity of hADSCs using immunostaining and RT-PCR analysis**. **a**, Cells were cultured for one week in selection media specifically designed for adipogenic differentiation. Adipogenic differentiation is shown with oil-red O staining and RT-PCR analysis for lipoprotein lipase. **b**, Cells were cultured for two weeks in selection media specifically designed for osteogenic differentiation. Osteogenic differentiation is shown with alkaline phosphatase staining and RT-PCR analysis for osteopontin and PTH receptor. **c**, Cells were cultured for three weeks in selection media specifically designed for chondrogenic differentiation. Chondrogenic differentiation is shown with Alcian blue staining and RT-PCR analysis for periecan and collagen type II. Scale bars measure 50 μm. GAPDH was used as a control. The intensity of each gene was normalized to GAPDH and these results were repeated at least five times. * *P *< 0.05, ** *P *< 0.01 compare with the primary hADSCs.

To determine whether hADSCs are pluripotent and able to differentiate into various cell types *in vitro*, hADSCs were cultured in selection media. Human ADSCs did not spontaneously differentiate during culture expansion. The differentiation of hADSCs into adipocytes, osteoblasts, and chondrocytes were also confirmed by specific staining and RT-PCR analysis (Figure 2). After one week of the adipogenic culture medium culture, more than 90% of the cells differentiated into lipid-laden cells that were stained with oil-red O. Lipoprotein lipase was markedly expressed in adipogenic cultured cells (Figure 2a). After two weeks of the osteogenic medium culture, the cells differentiated into osteoblasts, which were confirmed with strong alkaline phosphatase staining. The RNA expression of osteopontin in osteogenic cultured cells was higher than that of the control cells. Parathyroid hormone (PTH) receptor was also expressed in osteogenic cultured hADSCs, indicating differentiation into osteoblasts (Figure 2b). Differentiation of hADSCs into chondrocytes was determined by staining with Alcian blue, which identifies the proteoglycan extracellular matrix, a specific component of cartilage tissues. Compared to expression in the control cells, the relative RNA expression of periecan and collagen type II in chondrogenic cultured cells increased significantly (Figure 2c). These results demonstrate that hADSCs have an intrinsic plasticity regarding differentiation into various mesodermal lineage cells.

### Neural differentiation of hADSCs *in vitro*

**Figure 3:**
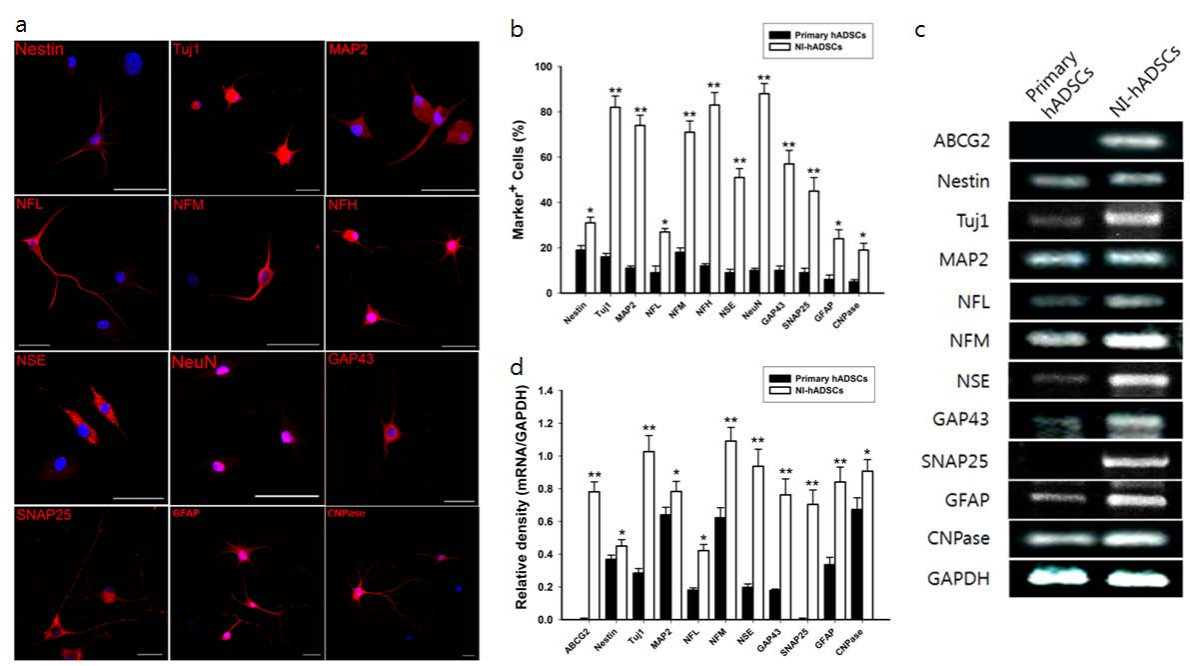
**Analysis of cell type-specific markers in NI-hADSCs**. Human ADSCs were induced to differentiate into neural cells in the presence of bFGF and forskolin over two weeks. **a**, Fluorescent immunocytochemistry revealed that the expression of nestin, Tuj1, MAP2, NFL, NFM, NFH, NSE, NeuN, GAP43, SNAP25, GFAP, and CNPase in NI-hADSCs increased more than those of primary hADSCs. Scale bars measure 50 μm. **b**, Immunocytochemical data depicts the high ratio of NI-hADSCs expression above neuronal markers. The number of positive cells was counted and the ratio of positive cells to nuclei was analyzed for each antigen (n = 7). **c **and **d**, RT-PCR analysis demonstrated increased mRNA expression for ABCG2, nestin, Tuj1, MAP2, NFL, NFM, NSE, GAP43, SNAP25, GFAP and CNPase genes. GAPDH was used as a control. The intensity of each gene was normalized to GAPDH and these results were repeated at least five times. * *P *< 0.05, ** *P *< 0.01 compare with the primary hADSCs

For neural induction, hADSCs were incubated with growth medium supplemented with bFGF for seven days, and then incubated in DMEM with forskolin for the next seven days. In neurally differentiated hADSCs, immunoreactivities for neural stem cell markers (nestin), neuronal markers (Tuj1, MAP2, NFL, NFM, NFH, NSE, and NeuN), synaptic markers (GAP43 and SNAP25), astrocyte marker (GFAP), and oligodendrocyte marker (CNPase) were very high when grown in the presence of bFGF and forskolin supplements (Figure 3a, 3b). After neural differentiation, the majority of NI-hADSCs exhibited distinct bipolar or multipolar morphologies with branched processes, which was well visualized by the immunocytochemistry of the somatodendritic marker, MAP2, and the axonal marker, GAP43 (Figure 3a). In basal hADSCs, the portion of hADSCs expressing cell type specific markers was very low. Marker-positive cells increased considerably with neural supplements. Following terminal differentiation with bFGF and forskolin, a large number of neural marker-positive neurons were observed in cultures when compared with that of GFAP-positive astrocytes and CNPase-positive oligodendrocytes (Figure 3b).

Reverse-transcription polymerase chain reaction analysis was used to monitor the extent of neural differentiation in mRNA levels of the stem cells. In NI-hADSCs, mRNA levels encoding for ABCG2, a neural stem cell marker, nestin, Tuj1, MAP2, NFL, NFM, NSE, GAP43, SNAP25, GFAP, and CNPase were higher compared with primary hADSCs (Figure 3c, 3d).

**Figure 4:**
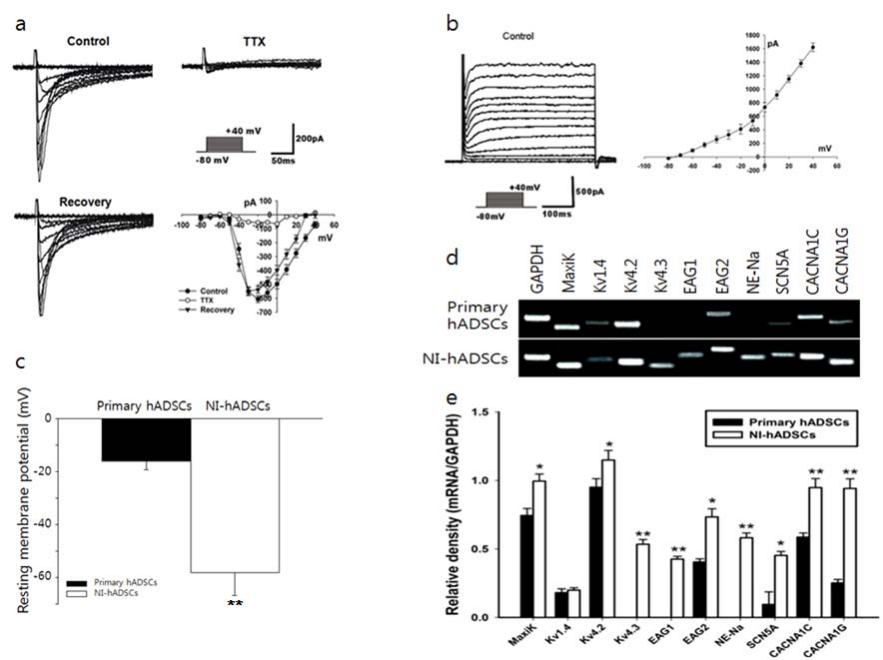
**Electrophysiological and RT-PCR analyses for ion channels in NI-hADSCs**. Electrophysiological features of the hADSCs demonstrated neuronal characteristics following neural differentiation. **a**, The holding potential was -80 mV and depolarizing steps were applied in mV intervals from -80 mV to +40 mV. Voltage-dependent sodium currents were activated from a depolarizing step of -30 mV and blocked reversibly by TTX 100 nM. The peak current-voltage relationship was plotted against the voltages, demonstrating the voltage-dependence of sodium currents (n = 16). **b**, The hADSCs grown with bFGF and forskolin demonstrated sustained outward potassium currents. The peak current-voltage relationship was plotted against the voltages, demonstrating the voltage-dependence of potassium currents (n = 12). **c**, Under current clamp conditions, the resting membrane potential of NI-hADSCs was more negative than that of control hADSCs grown without bFGF and forskolin (n = 16). **d **and **e**, The expression of molecular markers for ion channel subunits increased following neural differentiation in hADSCs. The mRNA expression of human large-conductance, voltage- and calcium-dependent K^+ ^channel marker, MaxiK; voltage-dependent K^+ ^channel marker, Kv1.4, Kv4.2 and Kv4.3; ether-á-go-go K^+ ^channel markers, Eag1 and Eag2; tetrodotoxin-sensitive Na^+ ^channel marker, NE-Na; voltage-dependent L-type Ca^2+ ^channel, alpha 1C subunit marker, CACNA1C; voltage-dependent T-type Ca^2+ ^channel, alpha 1G subunit marker, CACNA1G, and TTX-insensitive sodium channel marker, SCN5A, each increased in NI-hADSCs. GAPDH was used as a control. The RT-PCR assay was repeated five times independently of different cells and the representative data are shown. The intensity of each gene was normalized to GAPDH. * *P *< 0.05, ** *P *< 0.01 compare with the primary hADSCs.

To assess whether NI-hADSCs were differentiated into functional neurons, electrophysiological properties were tested in basal hADSCs and following neural differentiation using the patch clamp technique in whole-cell configuration. Primary hADSCs grown in the regular media in the absence of bFGF and forskolin were quiescent, exhibiting virtually no sodium current (n = 24, data not shown), which is responsible in both initiation and propagation of neural action potentials throughout the nervous system. However, following differentiation with bFGF and forskolin for two weeks, more than 75% of the NI-hADSCs expressed prominent voltage-dependent sodium currents up to 1.2 nA (18 of 24 cells). The mean peak amplitude of voltage-dependent sodium currents at -20 mV was -605 ± 36 pA (n = 18, Figure 4a). Sodium currents were blocked by 100 nM tetrodotoxin (TTX). Along with sodium current induction, hADSCs grown with differentiation factors demonstrated sustained outward currents, which exhibited voltage-dependence and kinetic characteristics for delayed rectifier potassium currents (Figure 4b). Under current clamp conditions, the resting membrane potential of neurally differentiated hADSC (-58.58 ± 8.46 mV, n = 24) was recorded to be significantly more negative than that of control hADSCs (-15.47 ± 5.13 mV, n = 24; *P *< 0.001, Fig. 4c).

In addition to functional studies, mRNA expression for ion channels related to outward and inward currents was investigated in primary- and NI-hADSCs with RT-PCR using specific primers (Table 2). In ADSCs, ion channel protein or gene expression has not yet been reported. The primary hADSCs expressed MaxiK (responsible for human large-conductance, voltage- and calcium-dependent K^+ ^channel), Kv1.4 and Kv4.2 (human voltage-dependent K^+ ^channel), Eag2 (human ether-á-go-go K^+ ^channel), SCN5A (TTX-insensitive Na^+ ^channel), CACNA1C (human voltage-dependent L-type Ca^2+ ^channel, alpha 1C subunit), and CACNA1G (human voltage-dependent T-type Ca^2+ ^channel, alpha 1G subunit) constitutively. However, primary hADSCs did not express Kv4.3 (human voltage-dependent K^+ ^channel), Eag1 (human ether-á-go-go K^+ ^channel), or NE-Na (TTX-sensitive Na^+ ^channel). Following neural differentiation with bFGF and forskolin, the gene expression levels of MaxiK, Kv4.2, Eag2, SCN5A, CACNA1C, and CACNA1G in NI-hADSCs were significantly higher than in the primary hADSCs (Figure 4d, 4e). Furthermore NI-hADSCs expressed Kv4.3, Eag1, and NE-Na. These results indicate that the hADSCs were effectively developed into functional neuron-like cells and thereby expressed neuron-specific phenotypes after being differentiated *in vitro*.

**Table 2:** Sequence of PCR primers

Gene	Sense	Antisense
Lipoprotein lipase	CTTCTGTTCTAGGGAGAAAGTG	TGCTGTGTAGATGAGTCTGATT

Osteopontin	GAAGGACAGTTATGAAACGAGT	AACATAGACATAACCCTGAAGC

PTH receptor	AACTACTACTGGATTCCTGGTGG	CTCCAAGATTTCTTGATCTCAG

Periecan	CATAGAGACCGTCACAGCAAG	ATGAACACCACACTGACAACC

Collagen type II	ACGGCGAGAAGGGAGAAGTTG	GGGGGTCCAGGGTTGCCATTG

ABCG2	CAAAACTTGCTGGGTAATC	ACAGAAACCACACTCTGACC

Nestin	CTCTGACCTGTCAGAAGAAT	GACGATGACACTTACAGAAT

Tuj1	CATGGATGCCGCTCAG	CAGGCAGTCGCAGTTTTCAC

MAP2	TGCCATCTTGGTGCCGA	CTTGACATTACCACCTCCAGGT

NFL	TCCTACTACACCAGCCATGT	TCCCCAGCACCTTCAACTTT

NFM	TGGGAAATGGCTCGTCATTT	CTTACTGGAAGCGGCCAATT

NSE	CTGATGCTGGAGTTGGATGG	CCATTGATCACGTTGAAGGC

GAP43	TCCTGAGCCCTGTCTCTCCCT	GCCACACTGTTTGACTTGGG

SNAP25	AGTTGGCTGATGAGTCGCTG	TGAAAAGGCCACAGCATTTC

GFAP	GCAGAGATGATGGAGCTCAATGACC	GTTTCATCCTGGAGCTTCTGCCTCA

CNPase	GGCCACGCTGCTAGAGTGCAAGAC	GGTACTGGTACTGGTCGGCCATTT

MaxiK	ACAACATCTCCCCCAACC	TCATCACCTTCTTTCCAATTC

Kv1.4	ACGAGGGCTTTGTGAGAGAA	CACGATGAAGAAGGGGTCAT

Kv4.2	ACCGTGACCCAGACATCTTC	CACTGTTTCCACCACATTCG

Kv4.3	GCCTCCGAACTAGGCTTTCT	CCCTGCGTTTATCAGCTCTC

EAG1	TGGATTTTGCAAGCTGTCTG	GAGTCTTTGGTGCCTCTTGC

EAG2	ACATCCTGCTTTTCGATTGG	CGGCTCTCTACCTGGCGTTG

NE-Na	GCTCCGAGTCTTCAAGTTGG	GGTTGTTTGCATCAGGGTCT

SCN5A	CCTAATCATCTTCCGCATCC	TGTTCATCTCTCTGTCCTCATC

CACNA1C	AACATCAACAACGCCAACAA	AGGGCAGGACTGTCTTCTGA

CACNA1G	CTGCCACTTAGAGCCAGTCC	TCTGAGTCAGGCATTTCACG

GAPDH	CATGACCACAGTCCATGCCATCACT	TGAGGTCCACCACCCTGTTGCTGTA

## Discussions

Our results demonstrate that ADSCs isolated from human earlobe fat express MSC-specific markers and can be differentiated into neural cells via bFGF- and forskolin-dependent pathways. In addition, NI-hADSCs have neural markers and functional neuron-like characteristics.

In the present study, the phenotypic expression of hADSCs is consistent over culture passages and the morphological features are the same as those previously reported [12,34,48,49]. Adipose tissue-derived stem cells are understood to express surface markers of CD9, CD10, CD13, CD29, CD44, CD49d, CD49e, CD54, CD55, CD59, CD73, CD90, CD105, CD146, CD166, and STRO-1, and lack hematopoietic lineage markers CD11b, CD14, CD19, CD34, and CD45 [20,34,50-53]. The hADSCs expressed MSC-specific cell type markers including CD13, CD44, CD90, and CD166, however, did not express CD14, CD34, and CD45 indicating that hADSCs in this study were of mesenchymal origin. Adipose tissue-derived stem cells also seem to possess the capacity to differentiate into multiple mesodermal lineages such as bone, fat, and cartilage [31,34]. The current study supports this hypothesis, characterizing the expression of multiple lineage-specific genes and proteins including adipocytes, osteoblasts, and chondrocytes. This observation has led us to speculate that adipose tissue may be a valuable source of mesodermal stem cells.

Reports indicate that the neural differentiation of ADSCs is achieved with different experimental protocols, Protocols include using chemical agents like β-mercaptoethanol[34,35], a mix of valproic acid, butylated hydroxyanisole, insulin, hydrocortisone [38,39,54], azacytidine [55,56], or a cocktail of isobutylmethylxanthine, indomethacin, and insulin [48,57,58]. A mixture of glial growth factors [36], a mixture including bFGF and platelet-derived growth factor [59], or brain-derived neurotrophic factor (BDNF) with retinoic acid [12] also evoked the neural induction of ADSCs *in vitro*. In addition, rodent ADSCs are differentiated into Schwann cell-like cells by a procedure that involves making floating neurospheres [40]. In bone marrow-derived MSCs, neural differentiation was induced via transfection of proneural genes [25], treatment with bFGF, forskolin and ciliary neurotrophic factor (CNTF) [9] or co-culture with neural cells [2,46]. In the present study, we used bFGF and forskolin to induce the neural differentiation of hADSCs because bFGF is known to generate neural precursor cells with a greater capacity for neuronal differentiation [9,26,60]. Contrary to bFGF, epidermal growth factor and cilliary neurotrophic factor are reported to restrict astrocyte lineages. Forskolin is a commonly used agent to increase the intracellular levels of cyclic adenosine monophosphate (cAMP) by activating the enzyme adenylyl cyclase. Furthermore, forskolin is reported to induce the neuron-like morphology and expression of NSE, NFH, and Tuj1 in human MSCs cultured in serum-free conditions [61,62]. Our experiments demonstrate that NI-hADSCs express increased immunoreactivities for neuronal markers Tuj1, MAP2, NFL, NFM, NFH, NSE, NeuN, GAP43, and SNAP25, as well as the increased mRNA expression of Tuj1, MAP2, NFL, NFM, NSE, GAP43, and SNAP25 compared to primary hADSCs (Figure 3), indicating that hADSCs differentiate into neural cells via bFGF and forskolin-mediated differentiation. Much like neuronal cells derived from other MSCs or embryonic stem cells [63,64], *in vitro*-transdifferentiated hADSCs also exhibited neuronal cell properties.

According to previous reports about neurogenic differentiation, ADSCs exhibit neuron-like morphology and express several proteins and genes consistent with the neuronal phenotype [20,35,54,57-59,65]. However, reliance on neural marker expression as an indicator of neurally differentiated MSCs has become unreliable because undifferentiated MSCs express several neural markers at both the mRNA and protein levels [42,66]. Adult mesenchymal stem cells constitutively express native immature neural proteins (nestin and Tuj1), whereas more mature neuronal and glial proteins (tyrosine hydroxylase, MAP2, and GFAP) are expressed in increasing passage numbers [67,68]. Undifferentiated ADSCs also express markers characteristic of neural cells such as NSE, vimentin, and NeuN [57]. In addition, inter-donor variability of expression of neural marker genes in MSC samples needs to be considered [42]. We therefore performed electrophysiological studies to investigate whether NI-hADSCs demonstrate the functional properties of mature neurons. Through patch-clamp recordings, NI-hADSCs were identified to generate prominent TTX-sensitive voltage-dependent sodium currents and outward potassium currents, both of which are hallmarks of mature neurons and crucial for signal transmission in the nervous system. In contrast to bone marrow-derived MSCs, in which combination of bFGF, forskolin, and CNTF was not sufficient to induce voltage-dependent sodium current [9], hADSCs expressed the sodium current by treatment of bFGF and forskolin. Furthermore, NI-hADSCs exhibited about -58 mV of resting membrane potential, indicating that they also have functional characteristics of neurons.

Recently, Anghileri *et al*. reported electrophysiological evidence of neuronal differentiation [12]. After differentiation with BDNF and retinoic acid, hADSC exhibited immunocytochemical evidence of neuronal differentiation in only 57% of cells and mean peak amplitude of approximately -189 pA for voltage-dependent Na^+ ^currents in differentiated hADSCs. Ashjian *et al*. also demonstrated that supplementation with isobutylmethylxnthine (IBMX), indomethacin and insulin induced transdifferentiation of human processed lipoaspirate cells into neuron-like cells [57]. However, they could not observe inward sodium currents. According to our immunocytochemical studies, our results indicate that more than 80% of hADSCs appear to differentiate into neuron-like cells under specific *in vitro *culture conditions with bFGF and forskolin, and express several proteins specific to the neuronal phenotype that exhibit neuronal morphology (Figure 3a, 3b). Furthermore, approximately 75% of hADSCs demonstrate neural differentiation properties under electrophysiological study, with about -605 pA of voltage-dependent sodium currents being recorded in NI-hADSCs. These results suggest that bFGF and forskolin may be more effective than BDNF, retinoic acid, IBMX, indomethacin, and insulin in inducing the neural differentiation of ADSCs.

Although ADSCs have been used for years in the investigation of cell replacement therapy and differentiation, information about ion channel expression remains undocumented. Undifferentiated bone marrow-derived hMSCs are known to express the TTX-sensitive sodium channel gene (NE-Na), potassium channel genes (MaxiK, Kv1.4, Kv4.2, Kv4.3, and Eag1) and the calcium channel gene (CACNA1C) [56,57]. However, this study indicates that primary hADSCs also express ion channel mRNAs, including potassium channel genes (MaxiK, Kv1.4, Kv4.2, and Eag2), calcium channel genes (CACNA1C and CACN1G), and the TTX-insensitive sodium channel gene (SCN5A), but do not exhibit the TTX-sensitive sodium channel gene (NE-Na) and other voltage-dependent potassium channel genes (Kv4.3 and Eag1) (Figure 4d). Since the primary hADSCs did not display voltage-dependent sodium currents prior to neural differentiation, gene expression results are consistent with electrophysiological data. However, neural induction with bFGF and forskolin increased the expression of these ion channel genes, particularly those expressed in the primary hMSCs, and induced three novel functional ion channel genes (NE-Na, Kv4.3, and Eag1), indicating the differentiation of hADSCs towards neuronal cells. This result is an initial finding on the expression of ionic channel genes in both primary- and NI-hADSCs.

## Conclusions

The present study demonstrates that hADSCs have the ability to act as mesenchymal stem cells and can effectively differentiate into functional neuron-like cells with bFGF and forskolin treatment.

## Methods

### Preparation of hADSCs

Fat tissue from the human earlobe was obtained from healthy donors 4-20 years of age. Informed consent was obtained from all donors according to the Guidelines of the Ethics Committee at Chonnam National University Medical School. Human mesenchymal stem cells were isolated from fat tissue samples according to the previous report [32]. Human fat tissue was rinsed with PBS containing 1% penicillin-streptomycin (Hyclone, Logan, UT, USA), cut into small pieces, and then incubated in a solution containing 0.075% collagenase type IA (Sigma Chemical Co., St. Louis, MO. USA) for 1 h at 37°C with vigorous shake. The top lipid layer was removed and the remaining liquid portion was centrifuged at 220 g for 15 min at room temperature. The pellet was treated with 160 mM NH_4_Cl (Sigma Chemical Co.) for 10 min to remove red blood cells. The remaining cells were filtered through a 40-μm nylon mesh filter (BD Falcon, Franklin Lakes, NJ, USA), and plated at a density of 1 × 10^5 ^cells in a 10-cm dish. Isolated human mesenchymal stem cells were grown as adherent cultures in Dulbecco's modified Eagle's medium (DMEM; Hyclone, Logan, UT, USA) supplemented with 10% fetal bovine serum (FBS; Hyclone) and 1% penicillin-streptomycin. After four weeks, cells were grown to approximately 80% confluence in a 37°C humidified incubator with a 5% CO_2 _and 95% air environment. The morphological features of the hADSCs were the same as those previously described [12,33]. The hADSCs used in the present study were from passages 3 to 7.

### Fluorescence-activated cell sorting

For fluorescence-activated cell sorter (FACS) analysis, hADSCs were harvested in trypsin containing ethylenediaminetetraacetic acid (Hyclone), washed twice with phosphate-buffered saline (PBS; Amresco, Inc., Solon, OH, USA), and stained on ice according to the manufacturer's recommendations with monoclonal antibodies (BD Biosciences PharMingen™, Heidelberg, Germany), including PE-CD13, FITC-CD14, FITC-CD34, PE-CD44, FITC-CD45, PE-CD90, and PE-CD166. At least 10,000 events were collected and analyzed with flow cytometry (BD FACSCalibur System, BD Biosciences Immunocytometry System, Heidelberg, Germany).

### Differentiation culture conditions

Adipogenic differentiation of hADSCs was induced by growing the cells in adipocyte differentiation basal medium containing an adipogenic supplement (Gibco BRL, Grand Island, NY, USA) for one week. One week following induction, cells were stained with oil-red O (Sigma Chemical Co.). Human fat tissue was used as a positive control. For negative controls, oil-red O was omitted from the reaction series. To induce osteogenic differentiation, hADSCs were cultured in an osteogenic differentiation basal medium containing osteogenic supplement (Gibco BRL). After two weeks, osteogenic differentiation was evaluated with alkaline phosphatase staining (TRACP & ALP double-stain kit, Takara Bio Inc. Shiga, Japan). Human bone tissue was used as a positive control. For negative controls, the substrate for alkaline phosphatase was omitted from the reaction series. For chondrogenic differentiation, cells were cultured for three weeks in the presence of chondrogenic supplement in chondrogenic differentiation basal medium (Gibco BRL). This medium was replaced every 3-4 days for three weeks. The development of chondrogenic differentiation was determined by staining the medium with Alcian blue (Sigma Chemical Co.). Human cartilage tissue was served as a positive control. For negative controls, oil-red O was omitted from the reaction series. To induce neural differentiation, hADSCs were grown in DMEM containing 1% FBS and supplementary 100 ng/mL basic fibroblast growth factor (bFGF, Invitrogen Co., Carlsbad, CA, USA) for seven days. The cells were then incubated in the presence of 10 μM forskolin (Sigma Chemical Co.) and over the next seven days, the cells were subjected to immunocytochemical, electrophysiological, and RT-PCR analyses.

### Reverse transcriptase-polymerase chain reaction (RT-PCR) analysis

To analyze the relative expression of different mRNAs, the amount of cDNA was normalized based on signals from the ubiquitously expressed GAPDH [69]. Total RNA was extracted from cultured cells by using TriReagent (Molecular Research Center, Inc., Cincinnati, OH, USA), and 1 μg of cDNA was reverse-transcribed using a reverse transcriptase (M-MLV, Gibco BRL) for 90 min at 42°C. PCR primer (Bioneer Co., Daejeon, Korea) pairs were selected to discriminate between cDNA and genomic DNA by using individual primers specific to different exons, when possible. The cDNA was amplified by 35 cycles of PCR (Takara Bio Inc.) using Ex-Taq polymerase (Takara Bio Inc.). Forward and reverse PCR oligonucleotide primers selected to amplify the cDNA are listed in Table 2. RT-PCR products were separated electrophoretically on 2% agarose gels (Sigma Chemical Co.).

### Immunocytochemistry

The immunocytochemical determination of cell type specific markers in hADSCs was performed as follows: Cells were grown on poly-L-lysine-coated aclar plastic coverslips for 10 days, fixed for 15 min with 4% paraformaldehyde (PFA; Sigma Chemical Co.), and blocked for 20 min with 0.5% Triton X-100 (Sigma Chemical Co.), which included 10% normal goat serum (Vector Laboratories, Inc., Burlingame, CA, USA) in PBS. Anti-nestin (1:300), anti-neuron-specific class III β-tubulin (Tuj1, 1:300), anti-microtubule associated protein-2 (MAP2, 1:200), anti-neurofilament-L (NFL, 1:300), anti-neurofilament-M (NFM, 1:300), anti-neurofilament-H (NFH, 1:300), anti-neuron specific enolase (NSE, 1:100), anti-neuronal specific nuclear protein (NeuN, 1:300), anti-growth associated protein 43 (GAP43, 1:300), anti-synaptosome-associated protein 25 kDa (SNAP25, 1:200), anti-glial fibrillary acidic protein (GFAP, 1:300), or anti-2',3'-cyclic nucleotide 3'-phosphodiesterase (CNPase, 1:100) were then added. Primary antibodies were incubated overnight at 4°C. After washing, Alexa 546-conjugated goat anti-mouse antibody (Molecular Probes, Invitrogen Co., CA, USA) was used as a secondary antibody and incubated at room temperature for 1 h. Nuclei were stained with DAPI for cell counting. Cells were observed using a Zeiss LSM510 confocal microscope (Carl Zeiss, Jena, Germany). All primary antibodies were purchased from Chemicon (Chemicon, Temecula, CA, USA). Experiments were performed in triplicate and the percentage of positive cells was randomly calculated. To perform quantitative analysis, the numbers of positive cells was counted on each acquired image by ImageJ1.42 (NIH), and the ratio to the number of nuclei was analyzed for each antigen. For negative controls, primary antibody was omitted from the reaction series. HB1.F3 human neural stem cells and postmortem human brain tissue were served as positive controls for nestin and the other antigens, respectively.

To stain the adipose cells with oil-red O, cells were fixed with 4% PFA for 15 min, washed with 60% isopropanol (Sigma Chemical Co.) and air-dried. A fresh 60% oil-red O working solution was prepared from stock solution (0.7 g oil-red O in 200 mL isopropanol) and filtered through a 45 μM syringe filter. Cells were stained with the working solution for 45 min and then washed five times with distilled water. During the alkaline phosphatase staining for osteoblasts, cells were fixed with 4% PFA for 15 min, washed with PBS, and incubated with alkaline phosphatase (using Takara kit) for 20 min. During Alcin blue staining for chondrocytes, cells were fixed 4% PFA for 15 min and then incubate with 3% acetic acid. Cells were stained with 1% Alcian blue in 3% acetic acid (pH 2.5) for 30 min and then washed with distilled water. The cells were examined using a light microscope (Olympus DP70, Japan).

### Electrophysiology

Cells grown on coverslips for two weeks were placed in a recording chamber on the stage of an inverted microscope (Eclipse TE 2000-S, Nikon; Tokyo, Japan), and voltage-dependent ionic currents and resting membrane potentials were recorded using the whole-cell patch clamp technique [70]. Patch micropipettes having a resistance of 2-4 MΩ were pulled with an electrode puller (PP830, Narishige, Japan) from borosilicate glass capillaries (G150T-3, Warner Instruments, CT, USA) and fire-polished using a microforge (MF-790, Narishige). Pipettes were filled with an intracellular-like solution containing 140 mM KCl, 5 mM NaCl, 1 mM CaCl_2_, 10 mM HEPES, 5 mM EGTA, 2 mM Mg-ATP for the inward Na^+ ^currents and outward K^+ ^currents to record. The pH was adjusted to 7.3 with KOH and filtered before use. The standard external solution was comprised of 140 mM NaCl, 5 mM KCl, 1 mM CaCl_2_, 1 mM MgCl_2_, 10 mM glucose, 10 mM HEPES. The solutions were adjusted to pH 7.3 with NaOH. Whole-cell currents were recorded at 22-24°C using a patch-clamp amplifier (Axopatch 200B, Axon Instruments, Foster City, CA, USA) and digitized by an analog-to-digital interface (Digidata 1320, Axon Instruments). Membrane currents were low-pass filtered at 2 kHz, sampled at 50 kHz, and then stored on the hard disk of an IBM-compatible computer using pClamp8.2 (Axon Instruments). The cells were held at -70 mV and depolarized in 10 mV intervals between -60 and +40 mV. The bathing or drug-containing solutions were applied to the recording chamber with a gravity-fed perfusion system.

### Statistics

Protein and mRNA levels were quantified by measuring the optical density of each band using computer-assisted densitometry (NIH Image analysis program, version 1.61). All values are expressed as the mean ± SEM. The one-way ANOVA test (Bonferroni *post hoc *comparison) was used to analyze differences between groups, with *P *< 0.05 being considered significant.

## Authors' contributions

SJ: Perform the main experimental work, collection and assembly of data, data analysis and interpretation, manuscript writing

H-HC: Proving of human tissue

Y-BC: Financial support

J-SP: Discussion and comment of the data and manuscript

H-SJ: Conception and design, data analysis and interpretation, manuscript writing, final approval of manuscript

All authors read and approved the final manuscript
